# Graph embedding ensemble methods based on the heterogeneous network for lncRNA-miRNA interaction prediction

**DOI:** 10.1186/s12864-020-07238-x

**Published:** 2020-12-17

**Authors:** Chengshuai Zhao, Yang Qiu, Shuang Zhou, Shichao Liu, Wen Zhang, Yanqing Niu

**Affiliations:** 1 College of Informatics, Huazhong Agricultural University, Wuhan, 430070 China; 2 School of Computer Science, Wuhan University, Wuhan, 430072 China; 3 School of Mathematics and Statistics, South-Central University for Nationalities, Wuhan, 430074 China

**Keywords:** lncRNA-miRNA interactions, Graph embedding, Ensemble learning, Attention mechanism

## Abstract

**Background:**

Researchers discover LncRNA–miRNA regulatory paradigms modulate gene expression patterns and drive major cellular processes. Identification of lncRNA-miRNA interactions (LMIs) is critical to reveal the mechanism of biological processes and complicated diseases. Because conventional wet experiments are time-consuming, labor-intensive and costly, a few computational methods have been proposed to expedite the identification of lncRNA-miRNA interactions. However, little attention has been paid to fully exploit the structural and topological information of the lncRNA-miRNA interaction network.

**Results:**

In this paper, we propose novel lncRNA-miRNA prediction methods by using graph embedding and ensemble learning. First, we calculate lncRNA-lncRNA sequence similarity and miRNA-miRNA sequence similarity, and then we combine them with the known lncRNA-miRNA interactions to construct a heterogeneous network. Second, we adopt several graph embedding methods to learn embedded representations of lncRNAs and miRNAs from the heterogeneous network, and construct the ensemble models using two ensemble strategies. For the former, we consider individual graph embedding based models as base predictors and integrate their predictions, and develop a method, named GEEL-PI. For the latter, we construct a deep attention neural network (DANN) to integrate various graph embeddings, and present an ensemble method, named GEEL-FI. The experimental results demonstrate both GEEL-PI and GEEL-FI outperform other state-of-the-art methods. The effectiveness of two ensemble strategies is validated by further experiments. Moreover, the case studies show that GEEL-PI and GEEL-FI can find novel lncRNA-miRNA associations.

**Conclusion:**

The study reveals that graph embedding and ensemble learning based method is efficient for integrating heterogeneous information derived from lncRNA-miRNA interaction network and can achieve better performance on LMI prediction task. In conclusion, GEEL-PI and GEEL-FI are promising for lncRNA-miRNA interaction prediction.

## Background

Non-coding RNAs (ncRNAs), including long non-coding RNA (lncRNA), miRNA, snRNA, are a category of RNAs that are not translated into functional proteins. A surge of studies has betrayed that ncRNAs have regulatory functions in biological processes [1–4]. LncRNAs are a class of ncRNAs with more than 200 nucleotides (nt), playing important roles in gene imprinting, immune response, and chromatin remodeling [1, 2]. MiRNAs are a category of single-stranded, endogenous, evolutionally conserved ncRNAs with 20-25 nt, which are involved in diverse biological processes, such as the regulation of metabolism, cell differentiation, gene expression, embryonic development, and apoptosis [3–5]. LncRNA-miRNA regulatory paradigms modulate gene expression patterns that drive major cellular processes (e.g., cell proliferation, cell differentiation, and cell death) which are central to mammalian physiologic and pathologic processes [6]. Furthermore, it has been found that both lncRNAs and miRNAs relate closely to severe diseases [7, 8]. Therefore, a critical key to reveal the mechanism of associated biological processes and diseases is to characterize various functions of lncRNAs and miRNAs.

LncRNAs and miRNAs produce complicated effects through their interactions with other biological molecules such as DNAs, RNAs, and proteins, thus conducting researches on lncRNA-biomolecule interactions contributes to portraying the functions of lncRNAs and miRNAs [9–11]. Lately, some studies have demonstrated that lncRNAs can be used as a decoy or sponge to regulate miRNAs’ behavior [12], indicating that identifying lncRNA-miRNA interactions (LMIs) helps to understand the functions of lncRNAs and miRNAs.

In earlier researches, unknown LMIs were identified through wet experiments. However, due to the laborious, costly, and time-consuming process of wet methods, it is more common to refine the candidate list in silico prediction for further validation experiments, in order to accelerate the identification of LMIs.

Recently, plenty of computational approaches have been proposed to predict LMIs. Huang et al. [13] propose a two-way diffusion model EPLMI for lncRNA-miRNA interaction prediction, which considers the known LMIs as a bipartite network. Huang et al. [14] develop GBCF, which builds a Bayesian collaborative filtering model using sequence, expression profiles, and target genes. Hu et al. [15] introduce a model, namely INLMI, which is based on the sequence similarity network and the expression similarity network. Zhang et al. [16] propose SLNPM which constructs the integrated similarity-based graph exploiting LMIs and genomic sequences, and implement a label propagation process on graphs for LMI prediction. These pioneers have produced good performances, but there still exist some limitations. On the one hand, some of the existing methods (e.g., EPLMI, GBCF and INLMI) heavily rely on biological features of lncRNAs and miRNAs, such as target gene information or expression profiles, which are not obtainable for all lncRNAs (miRNAs). On the other hand, the structure of the LMI network cannot be fully in pervious methods; nevertheless, it is fairly crucial to effectively utilize the structural and topological information of the LMI network for link inference.

Graph embedding learning (a.k.a. network representation learning), can be employed to preserve the structural property of the graph and map nodes of the graph into low-dimensional space, attracting widespread attention recently. To the best of our knowledge, some graph embedding methods have been exploited to reveal unknown associations between biomedical entities [17–19]. Motivated by the previous work in bioinformatics, we use graph embedding methods to capture information from LMI network.

Ensemble learning is one of the research hotspots in machine learning and pattern recognition. To date, ensemble learning methods have been increasingly used in computational biology because of their unique advantages in managing small samples, complex data structures, and high dimensionality [20]. Ensemble learning is an efficient technique that aggregates multiple machine learning models to achieve overall high prediction accuracy and good generalization [21]. It usually performs better than individual methods. Inspired by pioneering works [22–27], we adopt ensemble strategies to integrate individual predictions and embeddings to enhance the performance of LMI prediction.

In this paper, we propose novel LMI prediction methods based on graph embedding and ensemble strategies. Firstly, we calculate similarity based on lncRNA sequences and miRNA sequences and construct a heterogeneous network by combining them with the known LMIs. Secondly, we utilize five graph embedding methods (i.e., Laplacian Eigenmaps [28], HOPE [29], GraRep [30], DeepWalk [31], and GAE [32]) to capture structural information from the heterogeneous network, and learn the representation of lncRNAs and miRNAs. Later, we represent the lncRNA-miRNA pairs by merging lncRNA’s representation with miRNA’s representation, and build ensemble models based on pair features. As the extension of our previous work [33], we consider two ensemble strategies. For the former, we consider all the individual graph embedding based models as base predictors and integrate their predictions to develop a prediction method, named GEEL-PI. As for the latter, we construct a deep attention neural network (DANN) to learn lncRNA-miRNA pair representations by combining various graph embeddings, and develop a method, named GEEL-FI. The experimental results demonstrate that the proposed methods GEEL-PI and GEEL-FI can predict lncRNA-miRNA interactions with higher accuracy compared with other state-of-the-art methods. Moreover, the effectiveness of the prediction integration and attention network is proved by extensive experiments. Furthermore, we conduct case studies to validate the predicted LMIs which do not exist in our dataset. In conclusion, both GEEL-PI and GEEL-FI are useful for predicting LMIs. Our contribution can be summarized as:

1. We consider a variety of graph embedding methods to learn the embedded representations from the lncRNA-miRNA heterogeneous network.
2. We introduce a deep attention neural network to learn high-level sophistic representations by focusing on different aspects of embedded representations.
3. We consider two different ensemble strategies in this work. Then we design comprehensive experiments to compare them and analyze their effectiveness.

## Results and discussion

### Evaluation metrics

In this paper, we implement 5-fold cross-validation (5-CV) to evaluate our models. The following metrics are adopted in our experiments: the area under the precision-recall curve (AUPR), the area under the receiver-operating characteristic curve (AUC), F-measure (F1), accuracy (ACC), recall (REC), specificity (SPEC), and precision (PRE).

### Parameter settings

In this study, both GEEL-PI and GEEL-FI have two major components: graph embedding and ensemble learning. Here, we introduce parameter settings.

#### Parameter settings for graph embedding methods

In this study, both GEEL-PI and GEEL-FI adopt five graph embedding methods: LE, GraRep, HOPE, DeepWalk, and GAE to learn representations of lncRNAs and miRNAs. The graph embedding methods are implemented by BioNEV [19].

Here, we discuss the parameter settings of five graph embedding methods. Firstly, we fix the representation dimension of all the graph embedding methods *θ* as 120 and consider other specified parameters of each graph embedding method. For GraRep, we consider the *k* th transition probability matrix *k*-step ∈ {1, 2, 3, 4}. For DeepWalk, we fix the walk length *t* as 80, and consider the combinations of window size *w* ∈ {10,20,30,40} and walk per vertex *γ* ∈ {10,20,30,40}. For GAE, we consider autoencoder and variational autoencoder respectively, and select the size of hidden layers *β* ∈ {32,64,128,256,512,1024}. For the aforementioned graph embedding methods, we adopt the optimal parameters which achieve the highest AUPR scores.

#### Parameter settings for ensemble methods

In this paper, we propose two ensemble strategies: prediction combination for GEEL-PI and attention neural network for GEEL-FI. The detailed parameter settings are described below.

For GEEL-PI, Random Forest and Logistic Regression are implemented by “scikit-learn” [34] where default hyperparameters are adopted. For the logistic regression, we additionally adopt L2 regulation with default parameters.

For GEEL-FI, we tune the following parameter settings: (1) the number of hidden layers *μ* and the size of hidden layers *β* in DANN (2) the embedded representation vectors *ε* involved in the feature fusion (3) the dimension of lncRNA-miRNA pair features *θ* (4) the number of estimators *η* in Random Forest classifier.

In the attention layer of DANN, we design two groups of attention weights for individual lncRNA-miRNA pair features. For fully-connected layers, we consider different combinations of the parameters: number of hidden layers *μ* ∈ {1, 2, 3, 4}, size of hidden layers *β* ∈ {480, 240, 120, 60, 30}. Then we use the grid search to optimize these parameters according to their performances on 5-CV. Finally, we design a two-hidden-layer neural network, and the size of each layer is 120 and 60 respectively.

**Fig 1:**
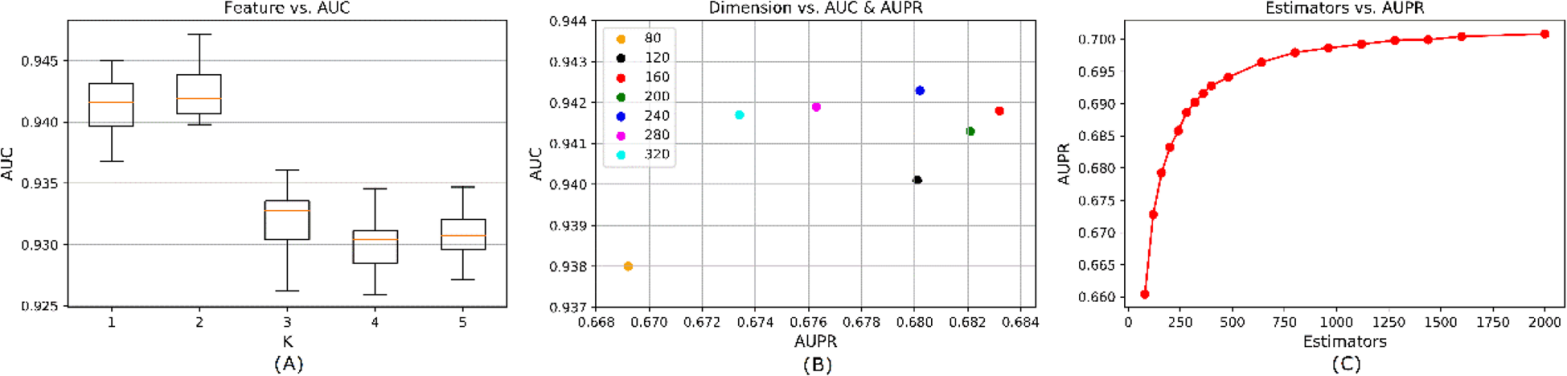
The influence of hyperparameters on performances of GEEL-FI model. **a** shows the box plot of AUC scores of GEEL-FI with different embedded representation integration. **b** shows the scatter plot of AUC and AUPR scores of GEEL-FI with different dimensions of lncRNA-miRNA pair embedded representations. **c** shows the line plot of AUPR scores of GEEL-FI with the different numbers of Random Forest estimators

As for the embedded representation vectors *ε*, we consider combinations of embedded representation vectors for merged lncRNA-miRNA pair features. For individual graph embedding methods, we implement 5-CV for 20 times. In the light of AUC and AUPR scores, we reorder five graph embedding methods as GraRep, LE, GAE, HOPE, DeepWalk. And then we select the top *K* features as the candidates for lncRNA-miRNA pair features. Here we visualize the trend of AUC scores over the combination of top *K* features in Fig. 1 (a). The fused feature based on the top 2 graph embedding methods (i.e. GraRep and LE) owns the best performances. Hence, we adopt *ε* = {*GraRep*, *LE*}.

We consider the dimension of lncRNA-miRNA pair features *θ* ∈ {80, 120, 160, 240, 280, 320} with the consideration of the AUPR and AUC scores. As presented in Fig. 1 (b), fused features of 160 dimensions have a higher AUPR score and that of 240 dimensions has a higher AUC score. In the subsequent experiment, pair features of 160 dimensions achieve better performance, thus we set *θ* = 160.

Eventually, we consider the number of estimators *η* in Random Forest from 80 to 2000. In Fig. 1 (c), when the number of estimators equals to 2000, the AUPR score has little improvement. Considering computational efficiency and time costs, we set *η* = 2000.

After analysis above, we adopt *μ* = 2, *β* = {240,120}, *ε* = {*GraRep*, *LE*}, *θ* = 160 and *η* = 2000 for GEEL-FI. All the parameters used in graph embedding ensemble methods are summarized in Table 1.

**Table 1:** Parameter settings for proposed methods

Methods	Components	Parameters
Graph embedding methods	Representation vector	dimension *θ*: 120
	GraRep	*k-step*: 1
	DeepWalk	walk length *t*: 80, walk per vertex *γ*: 30, window size *w*: 30
	GAE	variational Autoencoder, hidden size *β*: 512
GEEL-PI	Random Forest	default parameters
	Logistic Regression	L2 regulation with default parameters
GEEL-FI	DANN	hidden layers *μ*: 2, hidden size *β*: {240,120}
	Representation vector	embeddings *ε*: {*GraRep*, *LE*}
	Pair feature	dimension *θ*: 160
	Random Forest	estimators *η*: 2000

### Comparison with state-of-the-art methods

Here, we compare our models with several state-of-the-art methods including EPLMI [13], INLMI [15], and SLNPM [35]. EPLMI infers link probability according to the similarity between lncRNA and miRNA expression profiles. Specifically, EPLMI constructs a bipartite network using known lncRNA-miRNA interactions and exploits lncRNA (miRNA) expression profile information via the network for LMI prediction. INLMI integrates the sequence similarity and the expression similarity, and adopts a two-way diffusion algorithm to infer LMIs. SLNPM predicts LMIs by implementing a label propagation algorithm on two biomedical entities similarity graphs respectively. EPLMI and SLNPM are implemented according to the descriptions in the publications, then we evaluate the above models on our dataset by using 5-fold cross-validation experiments.

As shown in Table 2, GEEL-FI achieves the best AUPR score (0.7011), and the best AUC score (0.9578), and GEEL-PI achieves the second-best AUPR score (0.7004) and AUC score (0.9537), which significantly outperform other state-of-art methods. The substantial improvement of our models could be attributed to two factors: (1) GEEL-PI and GEEL-FI make the best of the structural properties implied in the lncRNA-miRNA heterogeneous network by employing graph embedding. (2) GEEL-PI and GEEL-FI adopt ensemble strategies (i.e. prediction integration and feature integration) to integrate multi-view information.

**Table 2:** Performances of different methods

Methods	AUPR	AUC	F1	ACC	REC	SPEC	PRE
EPLMI	0.0706	0.8494	0.1055	0.9939	0.1373	0.9962	0.0883
INLMI	0.0723	0.8477	0.1086	0.9935	0.1531	0.9956	0.0867
SLNPM	0.6207	0.9165	0.6652	0.9972	**0.6331**	0.9988	0.7016
GEEL-PI	0.7004	0.9537	**0.6933**	**0.9977**	0.5945	0.9995	0.8342
GEEL-FI	**0.7011**	**0.9578**	0.6915	**0.9977**	0.5790	**0.9996**	**0.8604**

**Fig 2:**
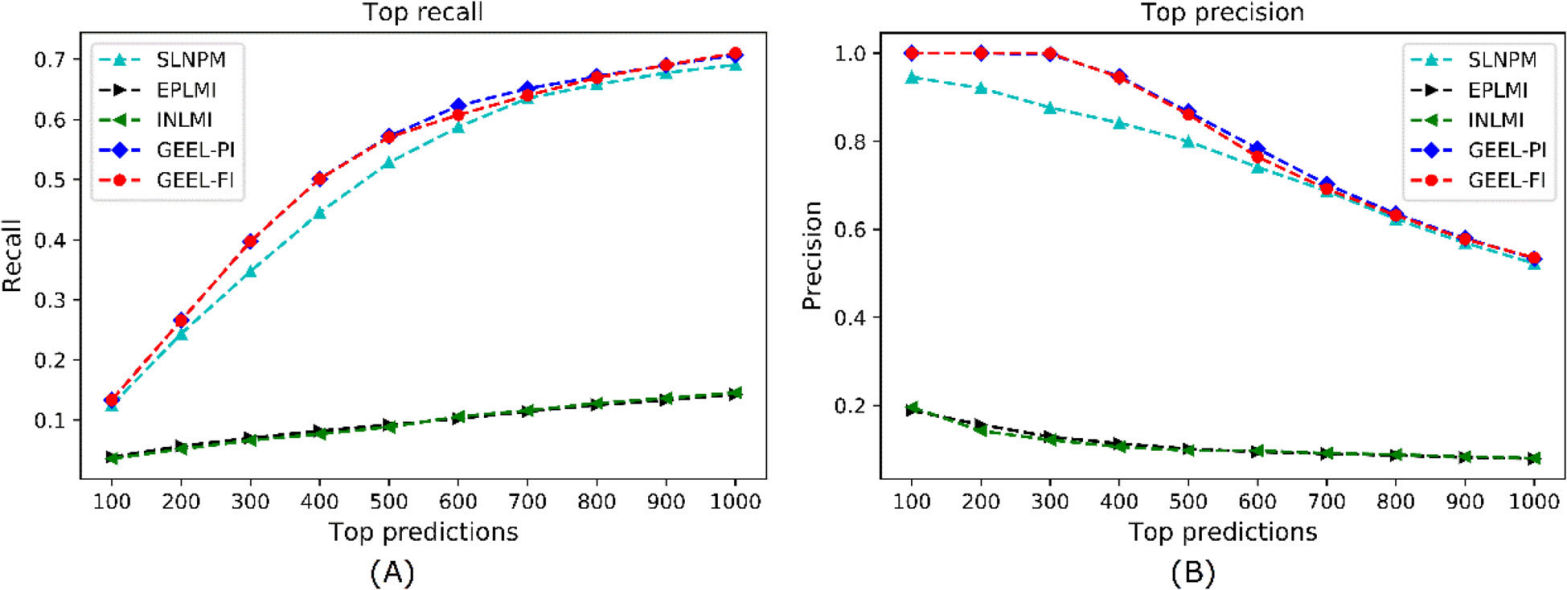
The top recall and top precision performances for different methods. **a** shows recall of different methods in top-ranked predictions. **b** shows precision of different methods in top-ranked predictions

In computational experiments, the top-ranked predictions are critical to reflect the performances of models. Here, we calculate the recall and precision of the aforementioned models on top-ranked predictions ranging from the top 100 to the top 1000. As presented in Fig. 2 (a), both GEEL-PI and GEEL-FI achieve best recall scores over all thresholds. For instance, when checking the top 500 predictions, GEEL-PI and GEEL-FI achieve recall scores of 0.5719 and 0.5706, nevertheless, the recall scores for SLNPM, EPLMI, INLMI remain 0.5283, 0.0921, 0.0884 respectively. Similarly, both GEEL-PI and achieve better precision scores than other benchmark methods as given in Fig. 2 (b). For example, both GEEL-PI and GEEL-FI can infer 86% real interactions in the top 500 predictions, whereas SLNPM, EPLMI, INLMI can only find 80, 10, 10% real interactions. Therefore, both GEEL-PI and GEEL-FI are preferable for LMI prediction compared with other state-of-the-art methods.

### Effect of ensemble learning

In this paper, we adopted two ensemble strategies to integrate heterogeneous information and develop our methods: GEEL-PI and GEEL-FI. In the following, we evaluate the performances of base predictors and our methods by 20 runs of 5-CV and discuss how the ensemble strategies improve performances.

As demonstrated in Table 3, generally, these graph embedding based models could produce satisfactory performances, achieving AUPR scores> 0.65 and AUC scores> 0.92. In terms of the standard deviations of 20 runs of experiments, all these prediction models could lead to stable results. The experimental results indicate that graph embedding methods can efficiently capture inherent properties from the lncRNA-miRNA heterogeneous network for LMI inference.

**Table 3:** Performances of based predictors and the ensemble models

Embedding	AUPR	AUC	F1	ACC	REC	SPEC	PRE
LE	0.6654 ± 0.0033	0.9430 ± 0.0017	0.6592 ± 0.0040	0.9976 ± 0.0001	0.5429 ± 0.0079	0.9995 ± 0.0001	0.8420 ± 0.0144
GraRep	0.6805 ± 0.0037	0.9417 ± 0.0019	0.6818 ± 0.0036	0.9977 ± 0.0001	0.5703 ± 0.0066	0.9996 ± 0.0001	0.8498 ± 0.0137
HOPE	0.6573 ± 0.0036	0.9281 ± 0.0022	0.6796 ± 0.0035	0.9976 ± 0.0001	0.5813 ± 0.0087	0.9994 ± 0.0001	0.8198 ± 0.0134
DeepWalk	0.6511 ± 0.0037	0.9383 ± 0.0018	0.6463 ± 0.0051	0.9974 ± 0.0001	0.5452 ± 0.0133	0.9994 ± 0.0001	0.7993 ± 0.0248
GAE	0.6664 ± 0.0031	0.9292 ± 0.0023	0.6754 ± 0.0033	0.9976 ± 0.0001	0.5666 ± 0.0086	0.9995 ± 0.0001	0.8395 ± 0.0185
GEEL-PI	0.7004 ± 0.0035	0.9537 ± 0.0022	**0.6933 ± 0.0032**	**0.9977 ± 0.0001**	**0.5945 ± 0.0063**	0.9995 ± 0.0001	0.8342 ± 0.0128
GEEL-FI	**0.7011 ± 0.0030**	**0.9578 ± 0.0013**	0.6915 ± 0.0029	**0.9977 ± 0.0001**	0.5790 ± 0.0063	**0.9996 ± 0.0001**	**0.8604 ± 0.0124**

Further, we integrate above five graph embedding based methods by ensemble strategies to enhance the accuracy of the model. GEEL-PI integrates different prediction scores from five graph embedding-based predictors, achieving AUPR score of 0.7004 and AUC score of 0.9537. GEEL-FI attentively integrates lncRNA and miRNA representations to obtain distinctive lncRNA-miRNA pair features, achieving AUPR score of 0.7011 and AUC score of 0.9578. Both GEEL-PI and GEEL-FI achieve superior performances compared with base predictors, which indicates our ensemble strategies can contribute to higher accuracy for LMI prediction.

To evaluate the generalization ability of our ensemble models, we design an experiment on different sparsity of the heterogeneous network by removal of a certain proportion of links. In the experiments, we randomly delete 10, 20, 30, and 40% of LMIs in the heterogeneous network. Then, we build the base predictors and the ensemble models on the networks with fewer interactions. Table 4 reports the AUPR scores of different prediction methods. As we can observe, the ensemble models GEEL-PI and GEEL-FI produce higher AUPR scores than all the base predictors as the ratios of removed links ranging from 10 to 40%. More importantly, when the network becomes sparser, the performances of the ensemble models are less affected than other individual predictors. For instance, when the number of removed interactions ranging from 10 to 20%, the AUPR scores of LE, GraRep, HOPE, DeepWalk, GAE, GEEL-PI and GEEL-FI reduce by 2.7, 2.1, 2.1, 2.3, 4.3, 1.7, and 1.7% respectively, which verifies the generalization ability and robustness of our ensemble models.

**Table 4:** Performances on the network of different sparsity

Removal ratio	LE	GraRep	HOPE	DeepWalk	GAE	GEEL-PI	GEEL-FI
10%	0.6496	0.6666	0.6448	0.6341	0.6537	**0.6858**	0.6838
20%	0.6323	0.6524	0.6311	0.6192	0.6254	**0.6744**	0.6719
30%	0.6124	0.6355	0.6171	0.5982	0.6206	0.6561	**0.6579**
40%	0.5884	0.6156	0.5959	0.5761	0.6009	0.6347	**0.6372**

In conclusion, integrating individual graph embedding based models with ensemble learning can effectively improve accuracy, generalization ability, and robustness in LMI prediction.

### Effect of attention network

In the design of GEEL-FI, we consider a deep attention neural network to integrate graph embeddings as the ensemble strategy. DANN learn lncRNA-miRNA pair features by capturing the different aspects of representation vectors. To validate the effectiveness of the attention mechanism, we evaluate the performances of GEEL-FI and our designed comparison method on LMI prediction.

**Fig 3:**
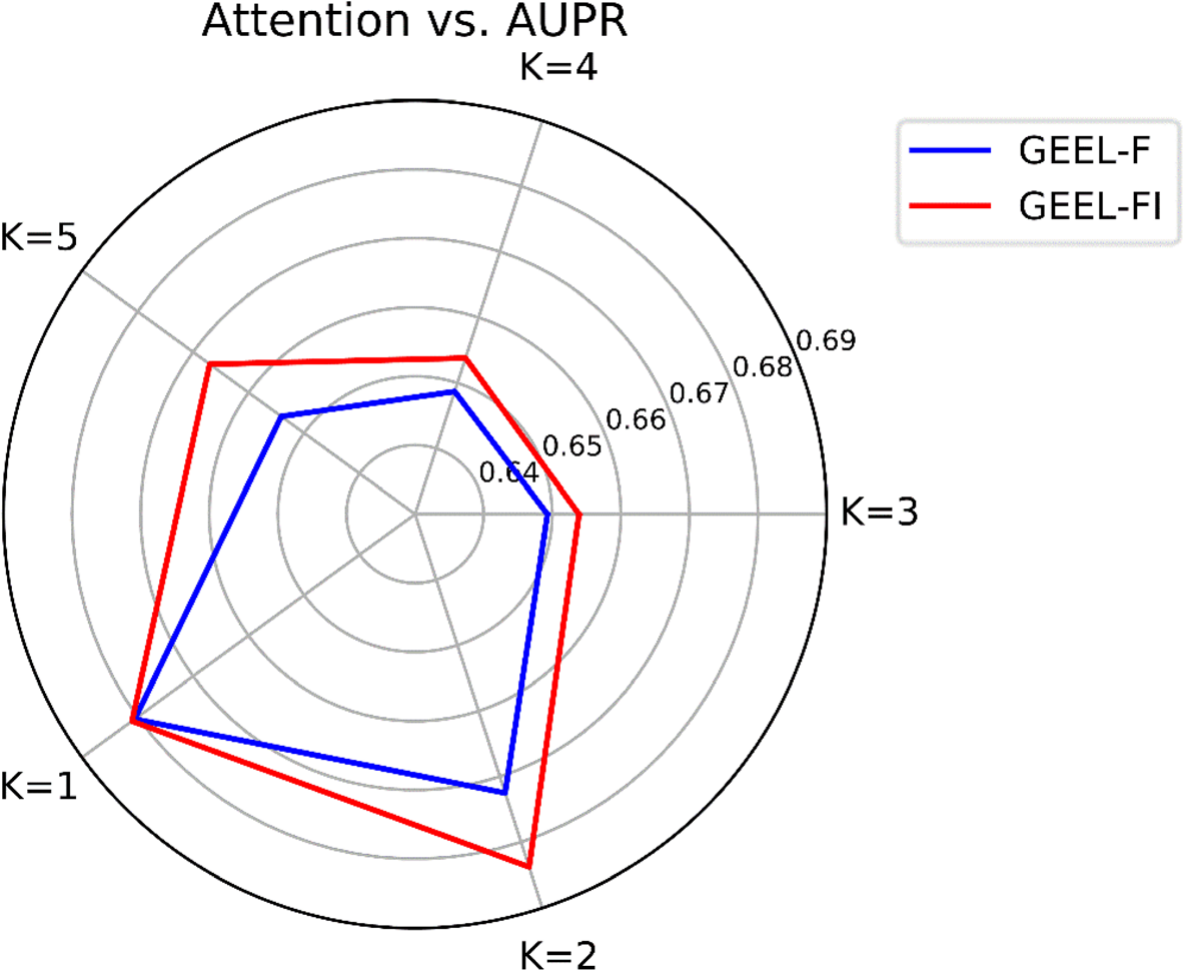
The AUPR scores of GEEL-F and GEEL-FI when different embeddings involved in feature fusion. GEEL-FI adopts attention mechanism to integration embeddings, GEEL-F does not

To validate the effect of attention network on feature fusion, we design the comparison variant as GEEL-F, which merges diverse embedded lncRNA and miRNA representations directly, without considering the different importance of embedded representations. For *i* th lncRNA and *j* th miRNA, the merged representation of lncRNA is defined as ${L}_i=\sum \limits_{k\in S}{l}_i^k$ and the merged representation of miRNA is defined as ${M}_j=\sum \limits_{k\in S}{m}_j^k$, where *S* is a set of lncRNA and miRNA representations learned by graph embedding methods. And the lncRNA and miRNA pair feature is computed as ***F***_***ij***_ = [*L*_*i*_; *M*_*j*_]. We construct GEEL-FI and GEEL-F based on learned graph embeddings. To validate the effectiveness of our attention mechanism at a larger scale, we choose the *K* embeddings for the fused feature. Here we respectively adopt *S* = {*GraRep*}, {*GraRep*, *GAE*}, {*GraRep*, *HOPE*, *DeepWalk*}, {*GraRep*, *HOPE*, *DeepWalk*, *LE*} and {*LE*, *GraRep*, *HOPE*, *DeepWalk*, *and GAE*} with respect to *K* = {1, 2, 3, 4, 5} as our benchmarks to compare the performances of GEEL-F and GEEL-FI for LMI prediction. As shown in Fig. 3, given *K* = {1, 2, 3, 4, 5}, GEEL-FI achieves AUPR scores of 0.6810, 0.6838, 0.6539, 0.6538 and 0.6670 which outperforms 0.6805, 0.6725, 0.6493, 0.6487 and 0.6541 respectively. The experimental result demonstrates the utilization of attention mechanism can contribute to better performance for LMI prediction. Therefore, we can conclude that our deep attention neural network can effectively merge multiple embedded lncRNA and miRNA representations and learn better lncRNA-miRNA pair features for LMI prediction.

**Fig 4:**
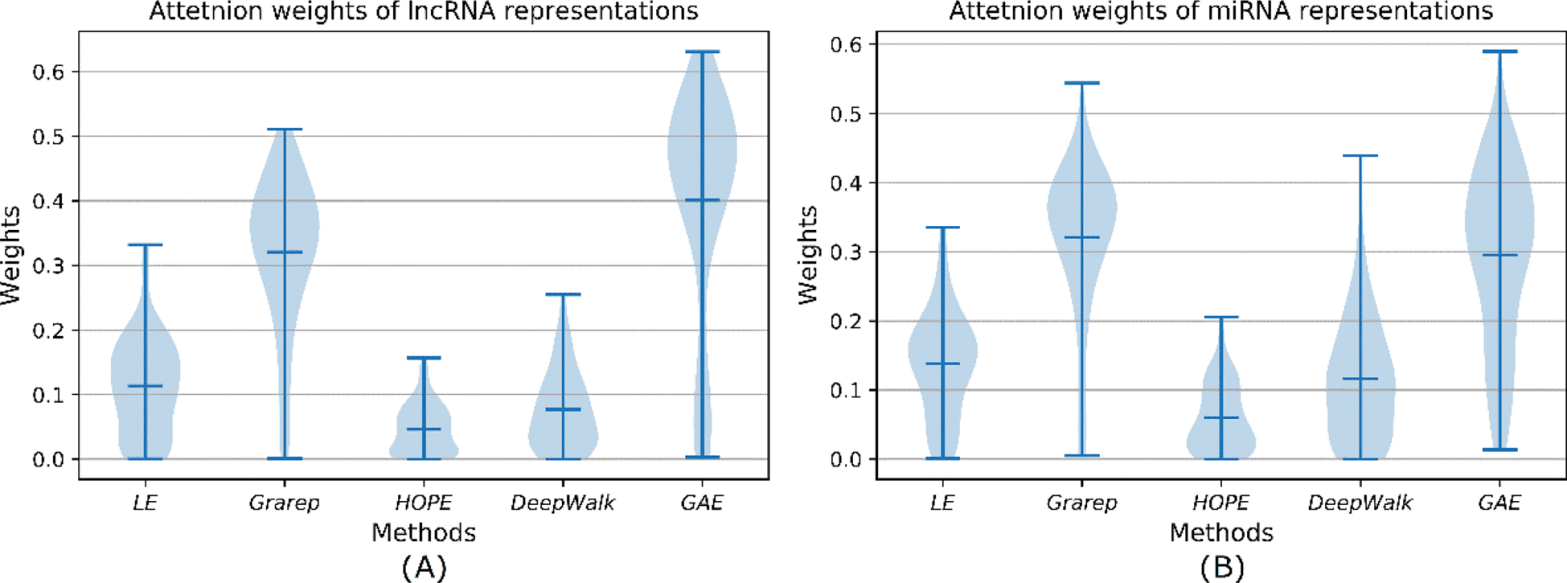
Attention weights in lncRNA and miRNA representations integration. **a** shows attention weights of lncRNA representations in GEEL-FI. **b** shows attention weights of miRNA representations in GEEL-FI

To further probe into how the attention network captures different aspects of embedded representations, we fix *K* as 5 and implement 5-CV for 20 times. Then we visualize the attention weights of lncRNA representations and miRNA representations learned by attention neural network. In Fig. 4, we can observe that (1) for lncRNAs, DANN generally pays much attention to the GAE-based embeddings, and for miRNA, it assigns higher attention weights to GraRep-based embeddings, which indicates the graph embedding based on neural network and matrix factorization method are efficient in LMI prediction. (2) furthermore, attention weights vary with lncRNA sequences and miRNA sequences in each fold, which validates DANN can adaptively adjust its attention to learn distinctive lncRNA-miRNA pair features according to specific lncRNA and miRNA data.

Consequently, our deep attention neural network can learn high-level sophistic representations of lncRNA-miRNA pairs and enhance the performances of GEEL-FI on LMI prediction.

### Case studies

The primary goal of computational methods is to refine the candidate list and guide further validation experiments. Here, we conduct case studies to demonstrate the practical capability of the proposed method for unknown LMI inference. Firstly, we train the model on our dataset. Then, we employ our model to score unlabeled lncRNA-miRNA pairs. Later, we validate the prediction result by a comprehensive datasets starBase [36]. Here, we list the top 10 LMIs in Table 5. As we can observe, both GEEL-PI and GEEL-FI can correctly infer 8 LMIs among their top 10 predictions. For instance, our proposed model can accurately predict that lncRNA lnc-ACER2–1:1 can interact with miRNA hsa-miR-106a-5p. ACER2 is one of the human alkaline ceramidases, and can produce lncRNA lnc-ACER2–1. MiRNA hsa-miR-106a-5p can participate in various biological processes, and are involved in severe diseases (e.g., gastric carcinoma and glioblastoma) [37, 38]. Some researchers have discovered that the expression of hsa-miR-106a-5p is down-regulated in breast tissues, and ACER2 could serve as a target gene of hsa-miR-106a-5p [39]. Whereas, the interaction between lnc-COL6A3–5:1 and hsa-miR-4500 is to be confirmed in the future. In general, both GEEL-PI and GEEL-FI are effective tools to indicate novel interactions between lncRNA and miRNA.

**Table 5:** Top 10 prediction of GEEL-PI and GEEL-FI

	GEEL-PI			GEEL-FI		
Rank	LncRNAs	MiRNAs	Evidence	LncRNAs	MiRNAs	Evidence
1	lnc-COL6A3–5:1	hsa-miR-4500	×	lnc-COL6A3–5:1	hsa-miR-4500	**×**
2	lnc-ACER2–1:1	hsa-miR-17-5p	√	lnc-ALYREF-1:1	hsa-miR-372-3p	**√**
3	lnc-FAS-1:1	hsa-miR-302b-3p	√	MIR17HG:2	hsa-miR-520a-3p	**√**
4	lnc-PDK3–1:1	hsa-miR-93–5p	√	lnc-PDK3–1:1	hsa-miR-302d-3p	**√**
5	lnc-ACER2–1:1	hsa-miR-106a-5p	√	USP2-AS1:10	hsa-miR-302b-3p	**√**
6	lnc-ALYREF-1:1	hsa-miR-372-3p	√	lnc-PDK3–1:1	hsa-miR-93–5p	**√**
7	MIR17HG:2	hsa-miR-520a-3p	√	lnc-NMRK1–1:1	hsa-miR-520d-3p	**√**
8	lnc-NMRK1:1	hsa-miR-520d-3p	√	lnc-ACER2–1:1	hsa-miR-17-5p	**√**
9	lnc-RPE-1:1	hsa-miR-130a-3p	×	lnc-ACER2–1:1	hsa-miR-106a-5p	**√**
10	lnc-PDK3–1:1	hsa-miR-302d-3p	√	lnc-RPE-1:1	hsa-miR-130a-3p	×

## Conclusions

LncRNAs and miRNAs are critical to cellular processes, and inferring their interactions contributes to betraying the mechanism of complicated disease. In this paper, we propose novel graph embedding ensemble learning methods: GEEL-PI and GEEL-FI. Comparison with other state-of-art methods demonstrates both GEEL-PI and GEEL-FI achieve higher accuracy performances for LMI prediction. The adoption of graph embedding methods overcomes the limitation of traditional features, and makes our model efficiently capture the inherent structural properties of LMI heterogeneous network. Further experiments indicate that ensemble learning and attention mechanism are powerful to enhance accuracy, generalization ability, and robustness of LMI prediction model. Moreover, the case studies are also performed to prove the practical capability of our methods. In conclusion, both GEEL-PI and GEEL-FI are promising for LMI prediction.

## Datasets and methods

### Datasets

We collect 8091 experimentally verified lncRNA-miRNA interactions from the lncRNASNP dataset [40]. After removing duplicated interactions, we obtain 5118 interactions between 780 lncRNAs and 275 miRNAs. We then download lncRNA sequences from NONCODE dataset [41] and miRNA sequences from miRBase dataset [42] separately. Ultimately, we compile our dataset with 3784 interactions between 642 lncRNAs and 275 miRNAs.

### Heterogeneous network

**Fig 5:**
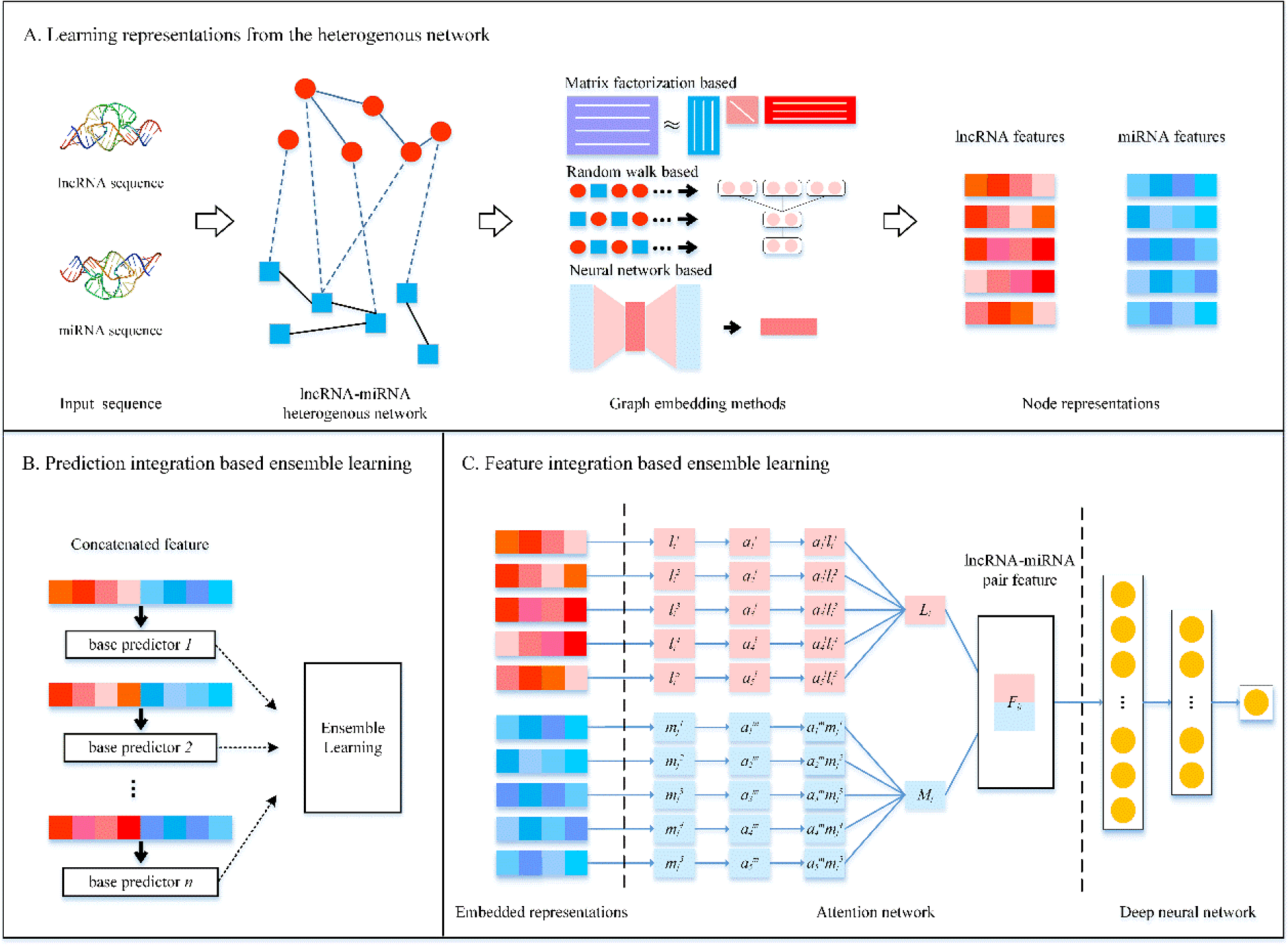
Flowchart of the proposed GEEL-PI and GEEL-FI. **a** by integrating the two similarity networks with the known lncRNA-miRNA interaction network, we construct a lncRNA-miRNA heterogeneous network. Different graph embedding methods are applied to the lncRNA-miRNA heterogeneous network to learn low-dimensional representations of lncRNAs and miRNAs. **b** for GEEL-PI, base predictors are trained based on the learned representations from different embedding methods. Then, their output predictions are integrated for further improving the performance and generalizability. **c** for GEEL-FI, by constructing a deep attention neural network, we integrate abundant embedded representation of lncRNA and miRNA to obtain distinctive lncRNA-miRNA pair features

To model the complicated relationship between biomedical entities, we design a lncRNA-miRNA heterogeneous network by integrating the known LMIs with the sequence similarity, as shown in Fig. 5 (a).

Given *r* lncRNAs and *t* miRNAs, the interaction matrix can be denoted by *A* ∈ *ℝ*^*r* × *t*^, where *A*(*i*, *j*) = 1 if *i* th lncRNA and *j* th miRNA are interacting, otherwise *A*(*i*, *j*) = 0. Our previous work [35] indicates that the pairwise similarity between biomedical entities (i.e. lncRNA and miRNA sequence similarity) can help to infer interactions. Therefore, same as our previous work, we extract *5*-spectrum feature [43] from lncRNA (miRNA) sequence and then calculate similarity by linear neighborhood similarity measure (LNS) [35]. In this way, we acquire lncRNA similarity matrix *S*_*l*_ ∈ *ℝ*^*r* × *r*^ and miRNA similarity matrix *S*_*m*_ ∈ *ℝ*^*t* × *t*^, where *S*(*i*, *j*) is the similarity score between *i* th and *j* th lncRNAs (miRNAs). Further, for a single biomedical entity, we consider the top 10 most similar entities as its immediate neighborhoods, and obtain adjacency matrix *W*_*l*_ ∈ *ℝ*^*r* × *r*^ and *W*_*m*_ ∈ *ℝ*^*m* × *m*^ from *S*_*l*_ and *S*_*m*_ separately. Ultimately, we regard biomedical entities (i.e. a lncRNAs and a miRNAs) as nodes and their relationships (i.e. LMs, lncRNA-lncRNA similarity and miRNA-miRNA similarity) as edges to construct the heterogeneous network *H*:

1 $$ H=\left[\begin{array}{cc}{W}_l& A\\ {}{A}^{\boldsymbol{T}}& {W}_m\end{array}\right]\in {\mathbb{R}}^{\left(r+t\right)\times \left(r+t\right)} $$

where *A*^***T***^ denotes the transpose of the matrix *A*.

### Graph embedding methods

To fully exploit the topological properties of the heterogeneous network, we choose graph embedding methods from three categories [19] (i.e. matrix factorization, random walk, and neural network).

From the matrix factorization-based category, we adopt Laplacian Eigenmaps (LE) [28], GraRep [30] and HOPE [29]. LE computes a low-dimensional representation of the dataset, optimally preserving local neighborhood information by using the Laplacian of the graph [28]. GraRep integrates global structural information of the graph into the learning process and learns high-order proximity [30]. HOPE can preserve high-order proximities of large scale graphs and is capable of capturing the asymmetric transitivity [29].

From the random walk-based category, We select DeepWalk [31]. DeepWalk uses local information obtained from truncated random walks to learn latent representations by treating walks as the equivalent of sentences [31].

We consider Graph Auto Encoder (GAE) [32] as a representative of the neural network-based methods. GAE obtains low-dimensional node representations by reconstructing the heterogeneous network with consideration of the first-order and second-order of proximities.

By employing the aforementioned graph embedding methods, the topological and inherent properties of the heterogeneous network are acquired, then the learned distinctive representations will be further used in the downstream task. as shown in Fig. 5 (a).

### Graph embedding ensemble learning based on prediction integration

In this section, we introduce a graph embedding ensemble learning method based on prediction integration (GEEL-PI). We build base predictors based on individual graph embedding methods, and further combine their predictions with ensemble strategy to infer LMIs.

To build a base predictor, firstly, we acquire the low-dimensional representations of miRNAs and lncRNAs using the corresponding graph embedding method. Then we denote lncRNA-miRNA pairs as the concatenation of two kinds of embeddings and further build a Random Forest predictor based on pairs. The reason why we adopt Random Forest lies in its high-efficiency.

Following the steps outlined above, we can construct five base predictors based on corresponding graph embedding methods. The five graph embedding methods are heterogeneous, which captures inherent structure properties from different aspects, thus they may demonstrate different generalization abilities on datasets. Therefore, it is natural to integrate several predictors by using ensemble strategies. Theoretically, ensemble learning is to build a model *ϕ* : (*f*_1_(*x*), *f*_2_(*x*), …, *f*_*n*_(*x*)) → {0, 1}, which maps the outcome of *n* base predictors to a label. Specifically, we consider logistic regression as the mapping function *ϕ*, which is simple but can model the nonlinear relationship between base predictors and labels. In this way, we construct GEEL-PI for LMI prediction as described in Fig. 5 (B).

### Graph embedding ensemble learning based on feature integration

In this section, we introduce a graph embedding ensemble learning method based on feature integration (GEEL-FI). We construct a deep attention neural network to learn lncRNA-miRNA pair representations, and further develop a classifier for LMI prediction.

The deep attention neural network contains attention layer and deep fully-connected neural layers, as given in Fig. 5(c). First, we consider attention mechanism to integrate different embedded representations. Because heterogenous lncRNA and miRNA features could be correlated and have redundant information, if directly merge them, it may affect the performances of conventional classifiers negatively. Attention mechanism can be used to assign importance weights to different representations which can determine the most relevant aspects, disregarding noise and redundancies in the input [44]. Motivated by its successful applications in many fields [45–51], we adopt an attention mechanism to integrate heterogeneous genomic representations. Then we consider the deep neural network (DNN) for feature refinement. DNN allows computational models with multiple processing layers to learn representations of lncRNAs and miRNAs with multiple levels of abstraction. Moreover, deep learning discovers intricate structure in large data sets by using the backpropagation algorithm to indicate how a machine should change its internal parameters that are used to compute the representation in each layer from the representation in the previous layer [52]. Therefore, we construct a DANN to adaptively capture the importance of each embedding feature and learn distinctive high-level representations for LMI prediction.

Specifically, given *i* th lncRNA and *j* th miRNA, by using five embedding methods, we obtain five lncRNA representations and five miRNA representations, let ${l}_i^k$ and ${m}_j^k$ (*k* = 1, 2, 3, 4, 5) denote embeddings from *LE*, *GraREP*, *HOPE*, *DeepWalk and GAE*, *i* = 1, 2, , …, *r* and *j* = 1, 2, , …, *t*. Then these representations are fed into attention networks. Let *L*_*i*_ denotes the integrated feature for *i* th lncRNA, and *M*_*j*_ denotes the integrated feature for *j* th miRNA. The merged representation of lncRNA and miRNA are defined as:

2 $$ {L}_i=\sum \limits_k{\boldsymbol{a}}_{\boldsymbol{k}}^{\boldsymbol{l}}{l}_i^k\kern1em $$

3 $$ {M}_j=\sum \limits_k{\boldsymbol{a}}_{\boldsymbol{k}}^{\boldsymbol{m}}{m}_j^k $$

where ${\boldsymbol{a}}_{\boldsymbol{k}}^{\boldsymbol{l}}$ denotes an attention weight measuring the importance of embedded representation *k* with respect to *i* th lncRNA, and ${\boldsymbol{a}}_{\boldsymbol{k}}^{\boldsymbol{m}}$ is an attention weight measuring the importance of embedded representation *k* with respect to *j* th miRNA.

Next, we concatenate *i* th lncRNA representation *L*_*i*_ and *j* th miRNA representation *M*_*j*_ to obtain lncRNA-miRNA pair feature ***F***_***ij***_, which indicates the interaction between *i* th lncRNA and *j* th miRNA:

4 $$ {\boldsymbol{F}}_{\boldsymbol{ij}}=\left[{L}_i;{M}_j\right] $$

where [*L*_*i*_; *M*_*j*_] is the concatenation of the two vectors.

To learn preferable representations of lncRNA-miRNA interactions, we consider the interacting lncRNA-miRNA pairs as positive instances and non-interacting lncRNA-miRNA pairs as negative instances to build a deep neural network. For *i* th lncRNA and *j* th miRNA, the lncRNA-miRNA pair feature ***F***_***ij***_ is fed into deep fully connected layers as following:

5 $$ {Z}_L= ReLU\left({\boldsymbol{W}}_{\boldsymbol{L}}\left( ReLU\left({\boldsymbol{W}}_{\boldsymbol{L}-\mathbf{1}}\cdots ReLU\left({W}_1{\boldsymbol{F}}_{\boldsymbol{ij}}+{\boldsymbol{b}}_{\mathbf{1}}\right)\right)+{\boldsymbol{b}}_{\boldsymbol{L}-\mathbf{1}}\right)+{\boldsymbol{b}}_{\boldsymbol{L}}\right) $$

where *L* denotes the number of hidden layers; *ReLU* is an activation function [53], and ***W***_***l***_ and ***b***_***l***_ are the weight matrix and bias vector for the *l* th layer, respectively.

And the prediction score between *i* th lncRNA and *j* th miRNA ${\hat{\rho}}_{ij}$ is computed as:

6 $$ {\hat{p}}_{ij}= Sigmoid\left(\boldsymbol{W}{Z}_L+\boldsymbol{b}\right) $$

where *Sigmoid* is an activation function; ***W*** and ***b*** are the weight matrix and bias vector, respectively.

And we adopt the following binary cross entropy as the loss function:

7 $$ \mathcal{L}=-\frac{1}{r\ast t}\sum \limits_{i=1}^r\sum \limits_{j=1}^t\left[{p}_{ij}\log {\hat{p}}_{ij}+\left(1-{p}_{ij}\right)\log \left(1-{\hat{p}}_{ij}\right)\right] $$

where $\mathcal{L}$ denotes loss function; *r* and *t* are total numbers of lncRNAs and miRNAs respectively. *p*_*ij*_ is a label, *p*_*ij*_ = 1 if *i* th lncRNA and *j* th miRNA are interacting, otherwise *p*_*ij*_ = 0;

Therefore, the attention weights ${\boldsymbol{a}}_{\boldsymbol{k}}^{\boldsymbol{l}}$ and ${\boldsymbol{a}}_{\boldsymbol{k}}^{\boldsymbol{m}}$ can be updated through the backpropagation algorithm [54] and gradient descent algorithm according to the above loss function $\mathcal{L}$. The update procedure can be described as:

8 $$ {\boldsymbol{a}}_{\boldsymbol{k}}^{\boldsymbol{l}}={\boldsymbol{a}}_{\boldsymbol{k}}^{\boldsymbol{l}}-\alpha \frac{\partial \mathcal{L}}{\partial {\boldsymbol{a}}_{\boldsymbol{k}}^{\boldsymbol{l}}} $$

9 $$ {\boldsymbol{a}}_{\boldsymbol{k}}^{\boldsymbol{m}}={\boldsymbol{a}}_{\boldsymbol{k}}^{\boldsymbol{m}}-\alpha \frac{\partial \mathcal{L}}{\partial {\boldsymbol{a}}_{\boldsymbol{k}}^{\boldsymbol{m}}} $$

where *α* is the learning rate of the neural network .

Here, to improve performances of LMI prediction, we build a Random Forest classifier based on pair features.

Therefore, we utilize DANN to integrate multiple features obtained by graph embedding methods to learn better representations of lncRNA-miRNA pairs, and construct GEEL-FI.

## Data Availability

The dataset lncRNASNP used in this study are freely available at http://bioinfo.life.hust.edu.cn/lncRNASNP. We download lncRNA sequence from NONCODE is now available at http://www.noncode.org. And the main datasets of miRNA sequences are available at http://www.mirbase.org.

## Abbreviations

GEEL-PI: Graph embedding ensemble learning based on prediction integration
GEEL-FI: Graph embedding ensemble learning based on feature integration
LMIs: lncRNA-miRNA interactions
nt: Nucleotides
DANN: Deep attention neural network
DNN: Deep neural network
5-CV: 5-Fold cross validation
AUC: Area under ROC curve
AUPR: Area under precision-recall curve
SEN: Sensitivity
SPEC: Specificity
PREC: Precision
ACC: Accuracy
F: F-measure
